# Risk Factors of Mental Health in University Students: A Predictive Model Based on Personality Traits, Coping Styles, and Sociodemographic Variables

**DOI:** 10.3390/medicina61091575

**Published:** 2025-08-31

**Authors:** Josefa A. Antón-Ruiz, Elisa Isabel Sánchez-Romero, Elena Cuevas-Caravaca, Miguel Bernabé, Ana I. López-Navas

**Affiliations:** 1 Faculty of Health Sciences, Universidad de Alicante (UA), 03690 Alicante, Spain; 2 Faculty of Education, Universidad Católica de Murcia (UCAM), 30107 Murcia, Spain; 3 Faculty of Psychology, Universidad Nacional de Educación a Distancia (UNED), 28040 Madrid, Spain; 4 Faculty of Medicine, Universidad Católica de Murcia (UCAM), 30107 Murcia, Spain

**Keywords:** anxiety, depression, stress, mental health, emerging adults, university students, risk factors, personality traits, coping styles

## Abstract

*Background and Objectives*: Data on mental health in university students have been increasingly concerning, with high prevalence rates of clinical conditions such as anxiety, stress, and depression. This study aims to evaluate the risk factors associated with mental health status and to develop a predictive model. *Materials and Methods*: A total of 242 university students were recruited (74.8% women). Participants’ ages ranged from 18 to 56 years (M = 25.81; SD = 7.59). Data collection were conducted through the Depression, Anxiety, and Stress Scale (DASS-21), the Big Five Inventory-10 (BFI-10), and the Coping Orientation to Problems Experienced Inventory (COPE-28). *Results*: Overall, mean scores across the three clinical dimensions are within the moderate range, but anxiety shows the highest mean value (M = 8.67, SD = 5.69) and is categorized as “extremely severe.” Additionally, identifying as female, living with family or roommates, and having high scores on passive coping styles were significant risk factors for mental health deterioration. In contrast, identifying as male, living with a romantic partner (cohabitation), and having high scores on the *Responsibility* personality trait were identified as protective factors against mental health impairment. *Conclusions*: Additional research is warranted to explore additional mediating variables and to develop specific intervention protocols for improving university students’ psychological well-being.

## 1. Introduction

A ‘risk factor’ in the field of mental health is operationally defined as any attribute, characteristic, or exposure that increases the likelihood of developing a mental health problem [1]. This definition is consistent with the main etiopathogenetic models that distinguish between causal, correlational, and moderating influences [2,3]. A risk factor increases the likelihood of developing a mental illness and can change throughout the lifespan. Some risk factors may be modifiable, while others may be genetic or biological. For this reason, recent research also highlights the role of genetic and epigenetic mechanisms (e.g., gene-environment interactions) and emerging environmental determinants, such as climate change, air pollution, and urbanization, which have been linked to a higher prevalence of mental health disorders [4,5] or the post-pandemic crisis [6].

The current global social, economic, and political landscape, shaped by the COVID-19 pandemic and its aftermath, has created a perfect scenario for an increase in psychological disorders, particularly among children, adolescents, and emerging adults, who have experienced a significant psychosocial impact in recent years [6,7,8,9,10,11,12,13,14]. In fact, several studies confirm that emerging adults exhibit higher rates of anxiety, stress, depression, and sleep disorders compared to other age groups, including adolescents [14,15,16]. This may be attributed to adolescents’ heightened sense of invulnerability, which leads them to somatize less in response to such threats [17]. Emerging adulthood is a transitional stage between adolescence and full adulthood, typically spanning ages 18 to 29 [18,19]. At this stage, individuals are neither adolescents nor fully adults, still grappling with a strong need for environmental adaptation, low self-confidence, and an undefined sense of personal goals, characteristic of adolescence. In addition, they now face significant life challenges, such as gaining access to higher education and navigating the competitive labor market [20,21]. Furthermore, it is worth noting that, in much of the world, university students constitute a substantial segment of the population, with most of them undergoing emerging adulthood [22]. Therefore, beyond the already concerning social context in which they live, they must also transition into and adapt to the demanding university environment—an entirely new setting where they continue to explore their identity [23]. This stage is also marked by a wide range of unfamiliar rights and responsibilities, such as major educational decision-making, changes in personal autonomy, and challenges related to family, financial, and social independence [24]. These circumstances are frequently associated with stress, anxiety, and depression [25]. Numerous studies report a diminished quality of life among university students, characterized by sleep difficulties, lack of motivation, and socio-affective problems linked to anxiety and depression. These conditions are often associated with poorly managed academic stress, the proximity to adolescence, romantic relationships, and financial instability [20,26,27,28]. Moreover, university students with psychological and/or psychiatric conditions are at greater risk of making unfortunate decisions that could negatively impact their academic performance and, consequently, the rest of their adult lives [29,30,31,32].

Regarding the vulnerability of this population, several studies highlight the protective role of psycho-emotional variables such as emotional intelligence and social support networks [33]. However, fewer studies have examined the role of personality traits in either predisposing individuals to or protecting them from mental health conditions and whether these traits directly influence the coping styles people adopt when facing overwhelming situations [20]. The Big Five personality model has been widely researched. Some of its dimensions are linked to healthy behaviors, while others are associated with maladaptive and psychosocially problematic behaviors in young populations. For example, low academic performance, academic burnout, delinquent behavior, substance use and abuse, and risky sexual behaviors have shown statistically significant associations with traits such as impulsivity, high neuroticism, and extraversion [20,34,35]. On the other hand, coping styles refer to the cognitive and behavioral strategies individuals develop to manage overwhelming demands in their environment, aiming to mitigate the psychological impact of stress [36]. According to the literature, active coping styles—focused on behavior and problem-solving—are associated with greater psychological well-being [37,38]. In contrast, passive coping styles—focused on emotional response and avoidance—tend to be linked to negative mood states such as anxiety and depression, thus posing a risk factor for mental health disorders [39,40]. Additionally, several studies confirm that the neuroticism dimension is associated with passive and maladaptive coping styles, whereas the conscientiousness dimension is linked to active coping strategies focused on behavior and problem-solving [37]. However, no significant evidence has been found regarding the remaining personality dimensions and coping styles. The relationship between personality and health has been studied within various theoretical frameworks, such as Eysenck’s personality model, stress control models, and healthy behavior models [41]. Nonetheless, no theoretical framework has yet fully explained the link between personality and health, with the clearest associations in disease contexts focusing on neuroticism and passive coping styles. Additional research is warranted to confirm preliminary indications that personality influences health in terms of its onset, persistence, and recovery [42].

It is essential to note that promoting and safeguarding mental health is a pressing challenge for higher education institutions. Ideally, universities should not only serve as spaces for professional training but also provide socio-affective support networks [43]. It is urgent to encourage research aimed at identifying potential risk factors to implement long-term mental health surveillance and support programs on university campuses, contributing to improved prevalence data [44]. When mental health issues remain unaddressed during youth, their consequences extend into adulthood, affecting both physical and mental health and limiting individuals’ prospects for leading a fulfilling life [6].

Given the concerns outlined above, the main objectives of this study are (1) to assess the mental health status of the participant sample; (2) to analyze the role of sociodemographic, personality, and behavioral variables as potential risk factors; and (3) to develop a predictive model tailored to the selected sample. Based on these objectives, the study proposes the following research hypotheses:

**H1.**  *A predominance of moderate to severe scores is expected across the three clinical axes: anxiety, stress, and depression.*

**H2.**  *Sociodemographic variables such as gender, age, marital status, living arrangements, and online study mode are expected to show statistically significant differences within the sample, allowing for the establishment of a risk/protective factor profile.*

**H3.**  *Certain personality traits—namely, neuroticism, openness to experience, and conscientiousness—are expected to explain moderate to severe anxiety, stress, and depression scores.*

**H4.**  *Passive stress coping styles are predicted to increase the likelihood of moderate to severe anxiety, stress, and depression.*

**H5.**  *The recruited sample is expected to confirm the ‘behavioral model of risk/health behaviors’ [45].*

## 2. Materials and Methods

### 2.1. Sample

During the sample recruitment phase, the following inclusion criteria were established: (1) being of legal age (18+) and (2) being enrolled in an undergraduate or postgraduate university program at the time of completing the questionnaire. The exclusion criteria were (1) failure to properly sign the informed consent form within the questionnaire; (2) being enrolled in a non-regulated higher education program; and (3) being enrolled at a university outside Spanish territory.

A total of 267 participants participated using incidental non-probabilistic sampling. After excluding 25 cases due to omissions and/or response errors, the final sample comprised 242 university students, with a completion rate of 90.6%. Of the total sample, 74.8% were women and 25.2% were men. The participants’ ages ranged from 18 to 56 years (M = 25.81; SD = 7.59).

### 2.2. Measures

A battery of tests was administered, consisting of the following:

#### 2.2.1. *Questionnaire ad hoc*

An ad hoc demographic questionnaire, including items on age, gender, marital status, children, employment status, living arrangements, type of studies, and study mode.

#### 2.2.2. *The Short Version of the Depression, Anxiety, and Stress Scales* (DASS-21) [46]

This instrument consists of three subscales—depression, anxiety, and stress—each containing seven items that assess the presence of symptoms experienced in the past seven days. The total scale comprises 21 items, with responses scored on a four-point Likert scale ranging from 0 (Did not apply to me at all) to 3 (Applied to me very much, or most of the time). The questionnaire is self-administered and can be completed in approximately three minutes. DASS-21 is derived from the original DASS-42, meaning that final scores are doubled. Normal score ranges are 0–9, 0–7, and 0–14, whereas high scores are 10–28, 8–20, and 15–34 for the depression, anxiety, and stress subscales, respectively. Regarding the reliability of the questionnaire, Cronbach’s Alpha of 0.95 was obtained in this research, exceeding reliability values reported in previous studies [47].

#### 2.2.3. *The Big Five Inventory-10* (BFI-10) [48]

The BFI-10 assesses global personality traits based on the “Big Five” model, with total scores calculated by summing the items within each domain separately to determine the most dominant trait. The dimensions included are extraversion (items 1 and 6); agreeableness (items 2 and 7); conscientiousness (responsibility) (items 3 and 8); neuroticism (items 4 and 9); and openness to experience (items 5 and 10). This inventory consists of 10 items rated on a five-point Likert scale (1 = Strongly agree, 5 = Strongly disagree) and is an abbreviated version of the 44-item Big Five Inventory (BFI-44). It uses two items per domain—one representing the positive pole and the other the negative pole—and takes approximately one minute to complete [49]. The BFI-10 has demonstrated good reliability and validity in large samples, and Cronbach’s alpha is not calculated due to the scale’s brevity and the fact that each dimension contains only two items [50].

#### 2.2.4. *The Coping Strategies Inventory (COPE-28)* [51]

This is an abbreviated version of the original Ways of Coping Checklist [52], measuring two coping styles: passive and active. Active coping signifies direct problem—solving, while passive coping seeks to avoid stressors and emotional engagement, providing short-term relief but maintaining negative symptoms through denial or cognitive avoidance [53]. It comprises 28 items across 14 dimensions, with two items per dimension, rated on a four-point Likert scale (1 = Never to 4 = Always). The scoring system involves summing items related to active and passive coping styles separately, with the higher score determining the individual’s predominant coping style. The questionnaire is self-administered, simple to complete due to its clarity, and requires approximately three minutes. The Cronbach’s alpha coefficient was 0.76 in this study, indicating a good level of reliability for measuring stress coping styles.

### 2.3. Procedure

This study employed a single-group ex post facto design with predictive purposes. Upon obtaining the approval of the University’s Research Ethics Committee (CEO62202), data collection was conducted through an online survey, distributed via social media and messaging services such as email, WhatsApp, and Telegram. The survey was designed using Google Forms and required approximately 10 min to complete. At the beginning of the questionnaire, participants were presented with an informed consent form detailing the study’s purpose, anonymity guarantees, voluntary participation, the right to withdraw, and compliance with General Data Protection Regulation (EU) 2016/679.

Once the data collection period ended, a data-cleaning process was carried out, eliminating cases in which participants had incomplete or incorrectly answered responses. Statistical analysis was conducted using IBM SPSS Statistics (version 29), except for the cross-validation procedure that was implemented in Python (version 3.9).

### 2.4. Data Analysis

First, frequency and descriptive analyses were conducted for qualitative variables, along with symmetry and kurtosis indices and the Kolmogorov–Smirnov goodness-of-fit test for normality for quantitative variables. These quantitative variables were described using standard central tendency measures (mean and median) and variability measures (observed range, standard deviation, and interquartile range). Secondly, to assess relationships between quantitative variables, the following correlation coefficients were used depending on the normality assumption: Pearson correlation coefficient (parametric) and Spearman correlation coefficient (non-parametric). Finally, a series of stepwise linear regression models were developed using forward variable selection. The model coefficients and goodness-of-fit estimators, expressed as R^2^ and adjusted R^2^ (*p* < 0.05), were included.

The order of variable inclusion in the stepwise regression models was guided by both empirical evidence and theoretical frameworks from prior research linking personality traits and coping styles with mental health outcomes. This approach sought to maximize model interpretability while retaining statistical parsimony [54].

In addition to the hierarchical regression models, we conducted a k-fold cross-validation procedure (k = 10) to evaluate the internal predictive stability of each model. Mean cross-validated R^2^ values were calculated for each dependent variable.

## 3. Results

### 3.1. Descriptive Statistics

**Figure 1:**
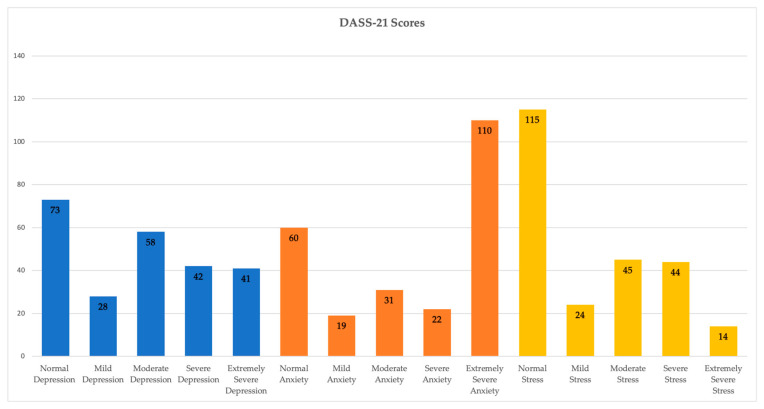
Frequencies of DASS-21 Scales’ Scores. Note: 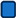 = Depression scoring ranges; 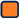 = Anxiety scoring ranges; 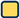 = Stress scoring ranges.

In Figure 1 and Table 1, descriptive data and frequencies of the dependent variables examined in this study can be consulted. The results obtained from the DASS-21 scale reveal significant variability in the levels of depression, anxiety, and stress among participants. As shown in Figure 1 and Table 1, the mean scores across the three clinical dimensions fall within the moderate range, with anxiety displaying the highest mean value (M = 8.67, SD = 5.69). Furthermore, normality tests indicate that these variables do not follow a normal distribution, thus supporting the use of non-parametric tests in certain inferential analyses. Statistically significant differences were found based on sociodemographic variables such as sex, marital status, and study modality, suggesting that contextual factors influence the manifestation of emotional symptoms.

### 3.2. Parametric and Non-Parametric Tests

In Table 2 and Table 3, parametric and non-parametric tests for qualitative variables examined in this study can be observed. Comparative analyses revealed significant sex-based differences, with women reporting significantly higher levels of depression (*p* = 0.038), anxiety (*p* = 0.023), and stress (*p* = 0.000) compared to men. Regarding occupational status, students presented higher scores across all clinical dimensions in comparison to employed and self-employed individuals. Furthermore, the non-parametric analysis confirmed that the variable ‘mode of study’ had a significant effect on stress levels (*p* = 0.036), with online students exhibiting higher levels than those attending in-person classes. Similarly, the analysis of cohabitation indicated that living with family or flatmates was associated with higher levels of depression and stress compared to those living with an affective partner or relative.

### 3.3. Correlations

Bivariate correlations were calculated to assess the relationships between mental health axes (depression, anxiety, and stress) and some quantitative factors, such as participants’ age, personality traits, and stress coping styles. Table 4 presents the correlation coefficients between these quantitative variables, showing that all dimensions of the ‘DASS-21’ questionnaire correlate directly and highly significantly with each other (*p* = 0.000). There is a significant inverse correlation between depression levels and responsibility (*p* = 0.036), indicating that more responsible individuals tend to exhibit lower levels of depression and vice versa. Additionally, there is an inverse correlation with age (*p* = 0.000), suggesting that depression levels decrease as age increases. Although significant, these correlations are of weak magnitude. A weak direct correlation is observed between passive stress coping and depression (*p* = 0.000), meaning that higher passive coping scores are associated with higher stress levels. Similarly, anxiety levels follow an identical correlation pattern to depression levels, correlating inversely with responsibility (*p* = 0.033) and age (*p* = 0.005) and directly with passive stress coping (*p* = 0.000). Likewise, stress levels correlate inversely with responsibility (*p* = 0.005) and age (*p* = 0.000) and directly with passive stress coping (*p* = 0.000).

### 3.4. Regression Models

Three models were developed to predict participants’ mental health axes in accordance with ‘DASS-21’, and they were based on sociodemographic factors, personality traits, and stress coping styles. Table 5 presents the variables selected for the predictive model of depression, along with their corresponding coefficients. This model includes, in order of importance, the individual’s age and sex, employment status, level of passive stress coping, cohabitation with a partner, level of active stress coping, and neuroticism. The model exhibits an adjusted R^2^ value of 0.180, indicating that the predictor variables can account for approximately 18.0% of the observed variability in depression levels. Since the model leaves the remaining 82.0% of the variability in the dependent variable unexplained, it is important to note that this value reflects a modest but meaningful explanatory capacity.

Table 6 presents the variables selected for the predictive model of anxiety scores, along with their corresponding coefficients. This model includes, in order of importance, the individual’s sex, level of passive stress coping, level of active stress coping, cohabitation with a partner, employment status, and level of neuroticism. As in the previous case, the model exhibits very limited predictive power, as it accounts for only 16.5% of the variability in anxiety levels (Adjusted R^2^ = 0.165).

Table 7 presents the variables selected for the predictive model of stress scores, along with their corresponding coefficients. This model includes, in order of importance, the level of passive stress coping, the individual’s sex, cohabitation with a partner, employment status, level of responsibility, type of studies pursued, and level of neuroticism. The model exhibits an adjusted R^2^ value of 0.183, indicating that the predictor variables account for only 18.3% of the variability in stress levels. Once again, this reflects a limited predictive power.

To further assess internal validity and address potential concerns about model generalizability, a k-fold cross-validation procedure (k = 10) was conducted for each model using all predictors. The mean cross-validated R^2^ values were 0.038 (SD = 0.219) for depression, 0.072 (SD = 0.161) for anxiety, and 0.064 (SD = 0.195) for stress. These values indicate a reduction in explained variance when models are applied to different subsets of the sample, highlighting the need for cautious interpretation of predictive performance.

## 4. Discussion

This analysis began by describing the sample based on its sociodemographic characteristics, mental health status, personality traits, and stress coping styles, concluding with the development of a predictive model for each symptomatic axis assessed by the DASS-21 (anxiety, depression, and stress).

Firstly, it is worth noting that normal and average scores predominated for the depression and stress axes in the recruited sample, whereas for the anxiety axis, the predominant score was “extremely severe.” Consequently, Hypothesis 1 of this study is only partially confirmed, as moderate/severe levels were expected across all three symptomatic axes measured by the DASS-21, as reported in previous similar studies conducted with university populations [6,9,10,12,13,14,16,20].

Secondly, it is important to highlight that sex has shown a clear trend across all three symptomatic axes, with women consistently scoring higher in anxiety, depression, and stress. Likewise, being childless, unemployed, or living with family (parents/guardians) or flatmates have all emerged as clear risk factors for poor mental health. In contrast, identifying as male, divorced, self-employed, and living with a partner appears to play a protective role against mental health disorders. Therefore, identifying as female has been identified as a risk factor that predisposes people to higher levels of depression, anxiety, and stress, as scores in these areas were significantly higher among female participants compared to their male counterparts, making sex a strong predictor [10,55]. Regarding age, an inverse relationship has been observed between age and levels of depression, anxiety, and stress in the sample. These findings suggest that as age increases, levels of these mental health conditions decrease. Therefore, Hypothesis 2 of this study is partially confirmed, as the expected relationship with sex aligns with previous national and international studies mentioned above, but the same does not hold for age. Additionally, it has been determined that the type of cohabitation significantly impacts depression levels, which are lower among individuals living with their partner. These findings suggest that being in a relationship may positively influence mental well-being. This may be explained by the fact that individuals living with a partner benefit from a broader socio-emotional support network compared to those living alone [33,55]. Furthermore, they have achieved one of the key milestones of early adulthood: independence from their family nucleus [18,24]. Regarding the field of study, it has been observed that this factor significantly affects stress levels, which are higher among undergraduate students and those studying online. This indicates that university students experience higher stress levels compared to those who are not currently studying. Thus, these results are highlighting the need for further investigation into this relatively unexplored association in the literature.

Furthermore, an inverse relationship has been found between responsibility scores and levels of depression, anxiety, and stress. This implies that individuals with higher levels of responsibility tend to exhibit better mental health compared to those with lower scores. Consequently, Hypothesis 3 of this study is partially confirmed, consistent with previous findings [37]. However, no significant results were found regarding neuroticism or openness to experience as risk factors, as suggested by other researchers [34,35].

A direct relationship has also been observed between passive stress coping and levels of depression, anxiety, and stress, indicating that individuals with a predominantly passive stress coping style are more likely to have poorer mental health indicators [39,40,52,56]. This confirms Hypothesis 4 of this study.

When interpreting the results, it is important to distinguish between factors supported by strong evidence for predicting mental health outcomes in college populations, such as gender, academic stress, and low social support, and those for which evidence is preliminary, such as certain personality traits and coping styles. This differentiation aligns the findings with broader trends in mental health research and highlights areas that require further investigation. While large-scale meta-analyses have found that low conscientiousness produces consistently strong effects on common mental disorders and that high neuroticism predicts the development of anxiety, depression, and substance abuse, the overall evidence on the role of personality traits and coping styles remains heterogeneous and developing, supporting the current classification of the evidence as preliminary [57]. Furthermore, a recent meta-analysis found that maladaptive, but not adaptive, coping strategies are significantly associated with greater psychological distress, reinforcing the idea that certain coping styles have greater empirical support as risk factors in mental health research [58]. While there is meta-analytic evidence linking traits such as low conscientiousness and high neuroticism with common mental disorders, the overall associations between traits and psychopathology remain heterogeneous and not fully consistent across all disorders [59].

Finally, it is noted that predictive models were developed during the analysis; however, they were found to have limited predictive power. Therefore, Hypothesis 5 of this study was not confirmed.

The inclusion of variables in the stepwise regression models followed a theoretically grounded sequence. Sociodemographic variables were entered first, given their well-documented association with mental health indicators in university populations [25,26], followed by personality traits (Big Five), particularly ‘Responsibility’ and ‘Neuroticism’, which have been linked to depression, anxiety, and stress [37,42]. Coping styles were included last, as they are often conceptualized as mediators or moderators between personality traits and mental health outcomes [41,52]. This hierarchical approach, widely used in psychological research, prioritizes the inclusion of stable, exogenous variables such as sociodemographics [59,60], followed by dispositional factors and more modifiable behavioral strategies. The sequence of regression blocks was informed by theory and prior empirical evidence [54], aiming to enhance interpretability while maintaining statistical parsimony.

Although the adjusted R^2^ values obtained in the hierarchical regression models (0.165–0.183) are modest, this range is consistent with the findings of similar studies examining psychological outcomes in international university populations. For example, some studies reported adjusted R^2^ values of approximately 0.164 when predicting mental health indicators in students, suggesting that the explanatory power of our models is comparable to that commonly found in the literature addressing this topic [56,61,62]. Comparing our results with previous studies reveals both convergence and divergence. The modest but significant contributions of personality traits and coping styles to mental health outcomes are consistent with the findings of similar research [60,61], where they also reported small to moderate effect sizes when examining these constructs alongside sociodemographic factors in university populations. However, unlike some studies conducted in different cultural contexts [62], our results showed a relatively stronger predictive role of coping strategies compared to personality traits in explaining anxiety and stress levels.

This discrepancy could be related to contextual factors specific to the post-pandemic academic environment in our sample, where adaptive coping mechanisms might have been particularly relevant in mitigating distress. Furthermore, the low proportion of explained variance, in line with previous research, underscores the importance of integrating additional variables such as social support, financial pressure, and academic load into future models to capture a broader spectrum of influences on mental health. The findings presented here are consistent with previous research indicating that sociodemographic characteristics, personality traits, and coping styles contribute, in unique but modest ways, to explaining the variability in mental health in young adult populations [59,60]. The relatively small proportion of explained variance underscores the multifactorial nature of mental health and the likely influence of additional variables not included in the current models, such as those mentioned above (e.g., academic stress, social support networks, and economic conditions).

In addition to the cross-sectional design, the modest cross-validation R^2^ values obtained using the k-fold procedure (0.038 for depression, 0.072 for anxiety, and 0.064 for stress) suggest that, while the models identify significant predictors in the current sample, their predictive capacity may be limited when applied to new data sets. This reinforces the need for future research to include external validation and, potentially, additional explanatory variables to improve the generalizability of the model.

To conclude, it is important to emphasize that these findings clearly highlight the urgent need for academic institutions to increase their involvement in supporting the mental health of university students [44]. These emerging adults represent a significant proportion of the general population [22], and most recent studies support the need to strengthen monitoring and intervention protocols to safeguard their psychological well-being [6,11,13]—an effort that should not rest solely on healthcare systems or private resources. Universities, like primary and secondary schools, are key socializing agents in the personal and social development of emerging adults. Therefore, they must take a proactive stance by expanding mental health prevention and intervention initiatives, addressing the specific factors that have been contributing to the exponential deterioration of student well-being since the pandemic and throughout the post-pandemic period [20].

## 5. Conclusions

This exploratory study has provided a detailed overview of the sociodemographic characteristics, mental health, personality traits, and stress coping styles of the analyzed sample. The findings highlight the importance of age, responsibility, gender, living arrangements, and type of study in participants’ mental health. The study also presents evidence of the protective effect of the personality trait ‘Responsibility’ against mental health issues, while emphasizing passive coping as a risk factor that should be considered. This trend can be explained by the fact that individuals with a predominant ‘Responsibility’ trait are more likely to engage in protective behaviors and health-related habits than the general population. Conversely, those who frequently adopt a passive coping style, characterized by problem avoidance, are more likely to develop anxiety-depressive symptoms. Furthermore, the need to develop more robust predictive models to better understand these phenomena is highlighted. Future studies should adopt more robust designs, such as multi-wave longitudinal follow-up across different semesters or academic years, to capture temporal variations in mental health. Furthermore, it is recommended to incorporate academic stressors, workload measures, and socioeconomic indicators as potential predictors. Mediation and moderation analyses could better elucidate indirect pathways between personality traits, coping styles, and mental health outcomes. External validation of the predictive models with independent datasets is also strongly recommended.

Another key limitation of this study is the need to increase male participation, as the sample lacked gender balance. Furthermore, in the three proposed models, greater predictive power could be achieved by binarizing the dependent variables, thus predicting the presence or absence of depression, anxiety, or stress.

All of these future lines of research would allow for improved methodological design, increasing the subsequent generalizability of results, and identifying potential variations related to the intrinsic characteristics of the academic calendar or the season, which would allow for better control of potential bias.

## Figures and Tables

**Table 1:** Descriptive data and normality tests.

Variables	*p*	m	M	Min/Max	SD	IQR
Depression	0.000 *	8.26	8.00	0.00/21.00	5.54	8.00
Anxiety	0.000 *	8.67	8.50	0.00/21.00	5.69	9.25
Stress	0.000 *	8.19	8.00	0.00/20.00	5.32	9.00
Extraversion	0.000 *	5.52	6.00	2.00/10.00	1.34	1.00
Agreeableness	0.000 *	5.90	6.00	2.00/10.00	1.48	2.00
Responsibility	0.000 *	5.88	6.00	2.00/10.00	1.33	2.00
Neuroticism	0.000 *	5.91	6.00	2.00/10.00	1.18	2.00
Openness	0.000 *	5.55	6.00	2.00/10.00	1.97	3.00
Active coping	0.093	19.66	20.00	4.00/31.00	4.80	7.00
Passive coping	0.000 *	22.38	22.00	11.00/34.00	4.20	5.00

* *p* significant value (variable doesn’t follow a normal distribution).

**Table 2:** Effect of sociodemographic variables on the clinical axes of depression, anxiety, and stress (parametric tests).

	Depression			Anxiety			Stress		
	*p*	m	SD	*p*	m	SD	*p*	m	SD
Sex									
Male (61)	0.000 *	6.54	4.97	0.000 *	6.51	5.16	0.010 *	6.18	4.86
Female (181)	0.038 *	8.85	5.61	0.023 *	9.40	5.69	0.000 *	8.87	5.30
Marital status									
Single (213)	0.000 *	8.35	5.54	0.000 *	8.70	5.73	0.000 *	8.24	5.31
Married (23)	>0.200	7.96	5.38	>0.200	8.78	5.55	>0.200	8.43	5.68
Civil partnership (3)	0.000 *	8.67	8.14	0.000 *	8.67	6.66	0.000 *	5.67	4.62
Divorced (3)	0.000 *	4.33	5.86	0.000 *	5.67	4.51	0.000 *	5.33	4.51
Children									
No (216)	0.000 *	8.38	5.57	0.000 *	8.72	5.71	0.000 *	8.23	5.29
Yes (26)	>0.200	7.35	5.25	>0.200	8.31	5.64	0.081	7.92	5.64
Occupation									
Student (167)	0.001 *	8.54	5.69	0.000 *	8.83	5.90	0.000 *	8.29	5.47
Salaried employee (62)	>0.200	7.15	4.63	>0.200	7.90	5.01	0.031 *	7.53	4.86
Unemployed (10)	>0.200	11.80	7.00	>0.200	12.20	5.51	>0.200	11.40	5.10
Self-employed (3)	0.000 *	4.33	0.58	0.000 *	4.33	1.53	0.000 *	5.67	2.31
Living arrangements									
With a partner (25)	0.008 *	5.32	4.92	0.060	6.32	4.68	0.006 *	5.92	5.29
With family (154)	0.018 *	8.64	5.27	0.024 *	8.78	5.57	0.000 *	8.32	5.04
With flat mates (49)	>0.200	9.20	6.49	0.092	9.76	6.48	0.010 *	8.82	6.36
Alone (14)	0.199	6.14	3.88	>0.200	7.93	4.97	>0.200	8.64	3.52
Studies									
Undergraduate (164)	0.000 *	8.46	5.55	0.000 *	8.98	5.76	0.000 *	8.66	5.26
Postgraduate (78)	>0.200	7.85	5.52	0.032 *	8.04	5.53	0.030 *	7.22	5.33
Study mode									
In-person (83)	0.008 *	7.25	5.13	0.008 *	8.02	5.54	0.000 *	7.70	5.25
Online (159)	0.005 *	8.79	5.68	0.007 *	9.01	5.76	0.001 *	8.45	5.35

* Significant, the variable does not follow a normal distribution. When n > 50, the Kolmogorov–Smirnov test is used for normality assessment, whereas for n < 50, the Shapiro–Wilk test is applied.

**Table 3:** Effect of sociodemographic variables on the clinical axes of depression, anxiety, and stress (non-parametric tests).

Variables	Test	Depression	*p*	Anxiety	*p*	Stress	*p*
		Statistic		Statistic		Statistic	
Sex	U de Mann–Whitney	2.75 **	0.006	3.4 **	0.001	3.42 **	0.001
Marital status	Kruskal–Wallis	1.7	0.638	0.89	0.827	1.58	0.664
Children	U de Mann–Whitney	−0.79	0.431	−0.34	0.730	−0.37	0.713
Occupation	Kruskal–Wallis	5.49	0.064	4.78	0.092	4.18	0.124
Living arrangements	Kruskal–Wallis	11.34 **	0.010	5.55	0.136	5.47	0.140
Studies	U de Mann–Whitney	−0.86	0.389	−1.22	0.221	−2.1 *	0.036
Study mode	U de Mann–Whitney	1.88	0.059	1.24	0.217	1.02	0.309

* Significant (*p* < 0.05); ** Highly significant (*p* < 0.01).

**Table 4:** Pearson (parametric) and Spearman (non-parametric) correlation matrix.

		DEP	ANX	STR	EXT	AGR	RES	NEU	OPE	ACT	PAS
ANX	Pearson			0.873 **							
	P-valor			0.000							
	Spearman			0.869 **							
	P-valor			0.000							
STR	Pearson	0.852 **	0.867 **								
	P-valor	0.000	0.000								
	Spearman	0.860 **	0.865 **								
	P-valor	0.000	0.000								
EXT	Pearson	−0.125	−0.098	−0.089							
	P-valor	0.051	0.128	0.169							
	Spearman	−0.102	−0.099	−0.073							
	P-valor	0.114	0.124	0.259							
AGR	Pearson	0.127 *	0.129 *	0.072	0.025						
	P-valor	0.048	0.045	0.264	0.695						
	Spearman	0.097	0.104	0.048	0.006						
	P-valor	0.133	0.105	0.455	0.926						
RES	Pearson	−0.119	−0.132 *	−0.172 **	0.277 **	0.108					
	P-valor	0.065	0.040	0.007	0.000	0.095					
	Spearman	−0.135 *	−0.137 *	−0.181 **	0.272 **	0.081					
	P-valor	0.036	0.033	0.005	0.000	0.208					
NEU	Pearson	0.130 *	0.133 *	0.104	0.089	0.268 **	0.165 **				
	P-valor	0.043	0.039	0.107	0.167	0.000	0.010				
	Spearman	0.109	0.091	0.073	0.136 *	0.217 **	0.167 **				
	P-valor	0.090	0.157	0.258	0.034	0.000	0.009				
OPE	Pearson	0.050	−0.035	−0.017	0.134 *	0.014	0.125	0.090			
	P-valor	0.442	0.585	0.797	0.037	0.828	0.053	0.161			
	Spearman	0.051	−0.032	−0.015	0.146 *	0.022	0.109	0.071			
	P-valor	0.432	0.622	0.812	0.023	0.731	0.092	0.270			
ACT	Pearson	−0.005	0.001	0.052	−0.029	−0.015	0.162 *	0.012	−0.119		
	P-valor	0.936	0.992	0.423	0.654	0.811	0.011	0.857	0.065		
	Spearman	0.026	0.043	0.091	−0.009	−0.038	0.176 **	0.046	−0.113		
	P-valor	0.684	0.502	0.159	0.885	0.555	0.006	0.475	0.079		
PAS	Pearson	0.201 **	0.214 **	0.221 **	−0.103	0.012	−0.050	−0.067	−0.155 *	0.612 **	
	P-valor	0.002	0.000	0.000	0.110	0.848	0.443	0.299	0.016	0.000	
	Spearman	0.230 **	0.239 **	0.243 **	−0.104	0.026	−0.036	−0.056	−0.147 *	0.589 **	
	P-valor	0.000	0.000	0.000	0.107	0.690	0.582	0.389	0.022	0.000	
AGE	Pearson	−0.225 **	−0.164 **	−0.166 **	0.114	0.023	0.182 **	0.128 *	0.105	−0.062	−0.208 **
	P-valor	0.000	0.010	0.010	0.077	0.726	0.005	0.046	0.102	0.333	0.001
	Spearman	−0.242 **	−0.180 **	−0.215 **	0.065	−0.005	0.150 *	0.139 *	0.063	−0.076	−0.202 **
	P-valor	0.000	0.005	0.000	0.317	0.943	0.020	0.031	0.325	0.241	0.002

* Significant (*p* < 0.05). ** Highly significant (*p* < 0.01). DEP: depression; ANX: anxiety; STR: stress; EXT: extraversion; AGR: agreeableness; RES: responsibility; NEU: neuroticism; OPE: openness; ATC: active coping; PAS: passive coping.

**Table 5:** Model 1: depression.

Variable	Coef.	Standardized Coef.	*t*	*p*
(Constant)	−1.01	-	−0.34	0.734
Age	−0.12	−0.17	−2.77 **	0.006
Sex	2.38	0.19	3.16 **	0.002
Employment status	4.22	0.15	2.59 **	0.010
Passive coping	0.40	0.31	4.03 **	0.000
Living arrangements	−2.90	−0.16	−2.67 **	0.008
Active coping	−0.24	−0.21	−2.84 **	0.005
Neuroticism	0.71	0.15	2.56 *	0.011
	R	R^2^	Adjusted R^2^	
Goodness of Fit Test	0.451	0.204	0.180	

* Significant (*p* < 0.05). ** Highly significant (*p* < 0.01).

**Table 6:** Model 2: anxiety.

Variable	Coef.	Standardized Coef.	*t*	*p*
(Constant)	−5.72	-	−2.06 *	0.04
Sex	2.92	0.22	3.75 **	0.00
Passive coping	0.49	0.36	4.88 **	0.00
Active coping	−0.28	−0.23	−3.13 **	0.00
Living arrangements	−2.95	−0.16	−2.68 **	0.01
Employment status	4.16	0.15	2.47 *	0.01
Neuroticism	0.64	0.13	2.24 *	0.03
	R	R^2^	Adjusted R^2^	
Goodness of Fit Test	0.432	0.186	0.165	

* Significant (*p* < 0.05). ** Highly significant (*p* < 0.01).

**Table 7:** Model 2: stress.

Variable	Coef.	Standardized Coef.	*t*	*p*
(Constant)	0.82	-	0.28	0.780
Sex	0.29	0.23	3.85 **	0.000
Passive coping	2.80	0.23	3.87 **	0.000
Active coping	−2.02	−0.12	−1.95	0.053
Living arrangements	4.86	0.18	3.09 **	0.002
Employment status	−0.77	−0.19	−3.20 **	0.002
Neuroticism	−1,91	−0.17	−2.82 **	0.005
	0.53	0.12	1.97 *	0.050
	R	R^2^	Adjusted R^2^	
Goodness of Fit Test	0.455	0.207	0.183	

* Significant (*p* < 0.05). ** Highly significant (*p* < 0.01).

## Data Availability

The original contributions presented in this study are included in the article. Further inquiries can be directed at the corresponding author.
